# Unilateral sudden hearing loss as the first presenting symptom of chronic myeloid leukemia: a case report and literature review

**DOI:** 10.3389/fonc.2025.1579765

**Published:** 2025-05-27

**Authors:** Le Sun, Yue Yin, Xiang Guo, Fan Yu, Jinkun Xu

**Affiliations:** ^1^ Department of Otorhinolaryngology-Head and Neck Surgery, Beijing Tsinghua Changgung Hospital, School of Clinical Medicine, Tsinghua University, Beijing, China; ^2^ Hematology Department, Beijing Tsinghua Changgung Hospital, School of Clinical Medicine, Tsinghua University, Beijing, China

**Keywords:** hearing loss, unilateral sudden hearing loss, sudden deafness, CML - chronic myelogenous leukemia, leukostasis

## Abstract

Chronic myeloid leukemia (CML) with sudden deafness as the first symptom is clinically rare and easily overlooked. No conventional treatment for CML-associated sudden hearing loss exists and hearing outcomes remain poor. Here, we describe a case in which sudden unilateral hearing loss was the first presenting symptom in a patient ultimately diagnosed with CML. The patient received imatinib mesylate, sodium bicarbonate, and allopurinol for CML, and systemic and local steroids, hemoreological therapy, and nutritional neurological drugs for hearing loss. Although the patient’s CML was effectively controlled, his hearing could not be restored. In the second part of the manuscript, we present the results of a systematic review of case reports of CML-associated sudden hearing loss, identified by screening PubMed, Web of Science, and Medline. Seventeen patients were identified. Hyperviscosity syndrome and labyrinthine artery occlusive infarction were the most common pathogenic mechanisms of hearing loss. In terms of auditory outcomes, eight patients showed no improvement, while nine demonstrated positive outcomes. Among those with improved hearing, four had undergone cochlear implantation, one received intrathecal methotrexate, and one underwent leukoreduction therapy. Aggressive cochlear implantation could improve hearing outcomes in cases where cochlear ossification has not taken place and the leukemia is controlled.

## Introduction

The relationship between leukemia and hearing loss was first described by Vidal in 1856 (1). Although otologic manifestations are observed in 15%–50% of leukemias (2), they are very rarely the first presenting features of the disease process. Chronic myeloid leukemia (CML) is a myeloproliferative disorder, which is characterized by the hyperplasia of hematopoietic cells, particularly those of the granulocytic lineage, resulting in hyperleukocytosis (i.e., a white blood cell [WBC] count > 100,000 cells/mm^3^) (3). Severe hyperleukocytosis can lead to hyperviscosity syndrome, in which the blood becomes too viscous for efficient circulation, which can result in a medical emergency called leukostasis. The pathogenesis of hearing loss in patients with leukemia is complex and includes four main areas: leukemic infiltration, infection, hemorrhage, and leukostasis. Vascular obstruction then results in tissue hypoxia and myocardial infarction (4). Infarction of the anterior inferior cerebellar artery (AICA) leads to ischemia of the inner ear, and ultimately, to hearing loss (5). Here, we describe a case in which sudden unilateral hearing loss was the first presenting symptom in a patient ultimately diagnosed with CML. In addition, we present the results of a systematic review of case reports and series documenting CML-associated hearing loss to better understand the underlying pathogenic mechanisms and identify the most effective therapeutic options for restoring hearing loss.

## Case presentation

**Figure 1:**
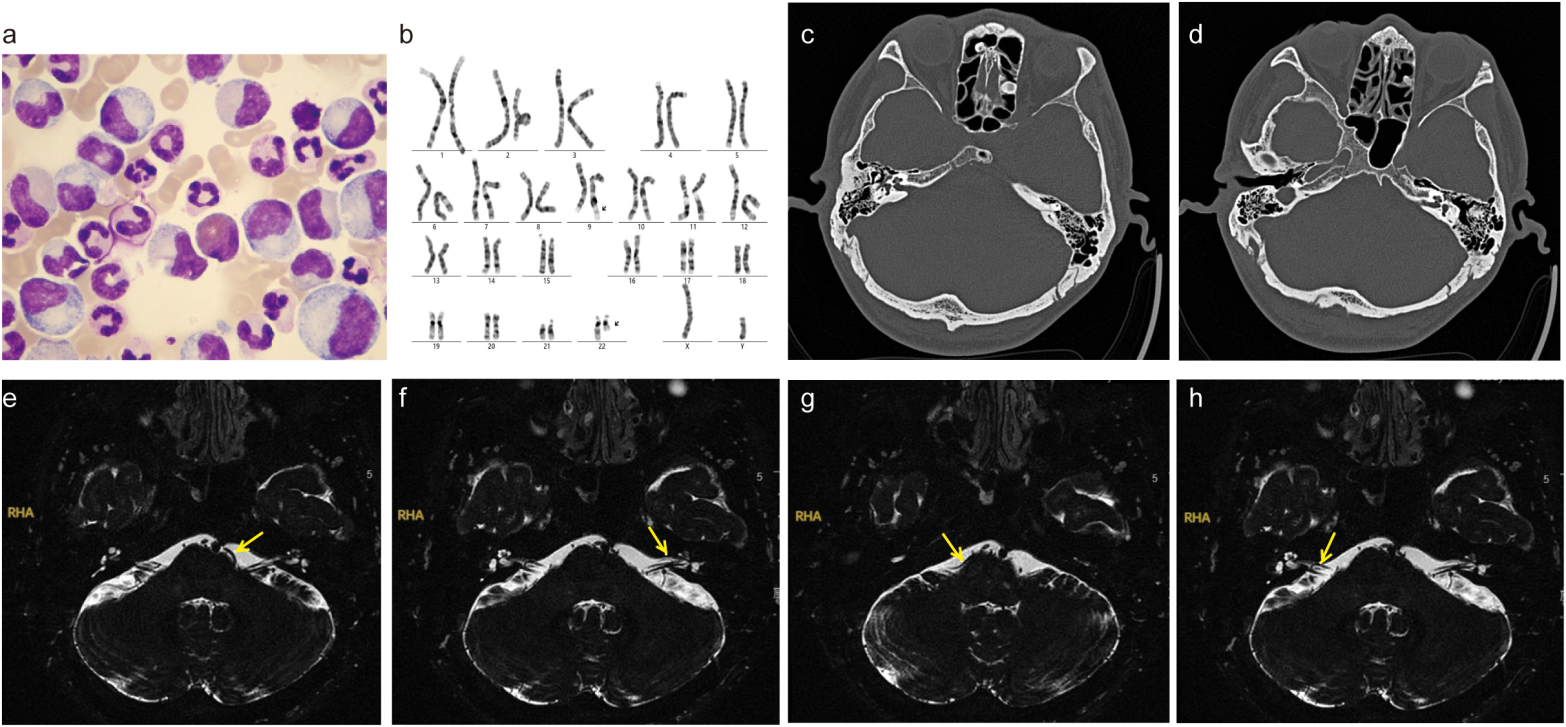
**(a)** A bone marrow cytomorphology image showing the extremely active proliferation of the granulocytic lineage (granulocytic lineage = 96%, erythrocytic lineage = 2%). **(b)** Chromosome karyotyping: 46, XY, t (9,22) (q34; q11). **(c, d)** Temporal bone computed tomography (CT) images showed no significant bilateral cochlear ossification. **(e, f)** Internal auditory canal magnetic resonance imaging (MRI) examination results; the yellow arrow indicates that the left anterior inferior cerebellar artery (AICA) was type IIB. **(g, h)** Internal auditory canal MRI examination results; the yellow arrow indicates that the right AICA was type IA.

**Figure 2:**
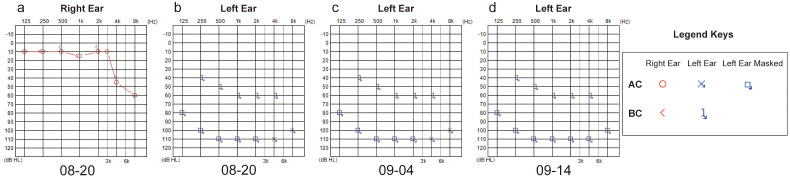
**(a, b)** Pre-treatment binaural hearing. **(c, d)** Post-treatment left-ear hearing.

A 43-year-old man presented to the emergency department of our hospital in August 2024 with unilateral (left) sudden hearing loss, vertigo, and nausea lasting 2 days. He had no prior history of hearing loss, tinnitus, or vertigo and denied any previous otologic trauma, drug use, noise exposure, or upper airway infection incidents. The initial examination revealed that the patient had hepatosplenomegaly; however, his vital signs were normal. A routine blood test in the emergency department revealed that the patient had leukocytosis, with a WBC count of 311 × 10^9^ cells/L. Subsequent bone marrow examination in the hematology department showed evidence of chronic-phase CML with 94.76% BCR-ABL1 (P210) positivity (**Figure 1a**). The results of the chromosomal karyotype test were also abnormal; the karyotype was 46, XY, t (9,22) (q34; q11) (**Figure 1b**). Assessment at our department revealed that the patient’s external auditory canal and tympanic membranes were both normal. However, the audiometric examination showed that he had complete sensorineural hearing loss (SNHL) in the left ear (**Figures 2a, b**). The patient’s temporal bone computed tomography (CT) and cranial magnetic resonance imaging (MRI) results were normal (**Figures 1c, d**). An MRI examination of the internal auditory canal (IAC), however, revealed that the branching pattern in the cerebellopontine angle (CPA) area of the left AICA (6) was type IIB, while that of the right AICA was type IA (**Figures 1e–h**).

**Figure 3:**
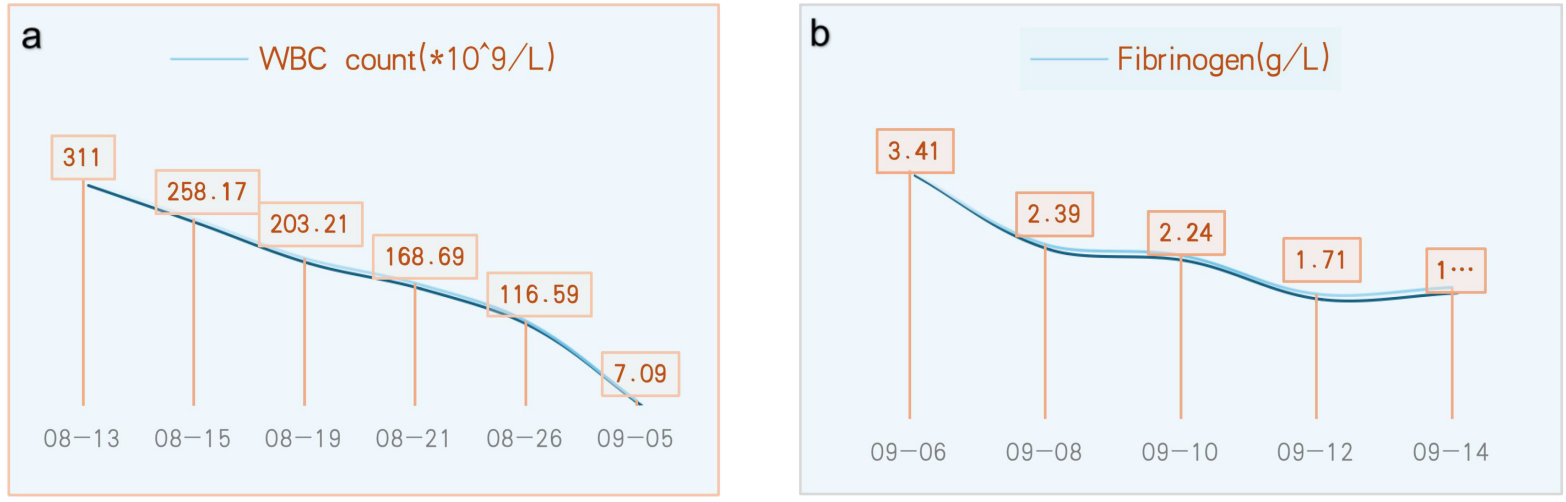
Monitoring of the WBC count (× 10^9^ cells/L) **(a)** and fibrinogen levels (g/L) **(b)**.

After 22 days of treatment with imatinib mesylate, sodium bicarbonate, and allopurinol for CML, the patient’s WBC count returned to normal (7.09 × 10^9^ cells/L) (**Figure 3a**). The patient continued to take daily imatinib mesylate until now following discharge and underwent a 5-day intravenous infusion of methylprednisolone to treat hearing loss. After a follow-up hearing test revealed no improvement, intratympanic steroid (methylprednisolone) was administered five times (on alternate days) into the left ear under local anesthesia (**Figure 2c**). After assuming that fibrinogen was being monitored greater than 1.0 g/L, administer five injections of batroxobin on alternate days (**Figure 3b**). Although the tinnitus and vestibular symptoms were alleviated, the hearing capacity of the left ear had not significantly improved (**Figure 2d**).

## Methods

**Figure 4:**
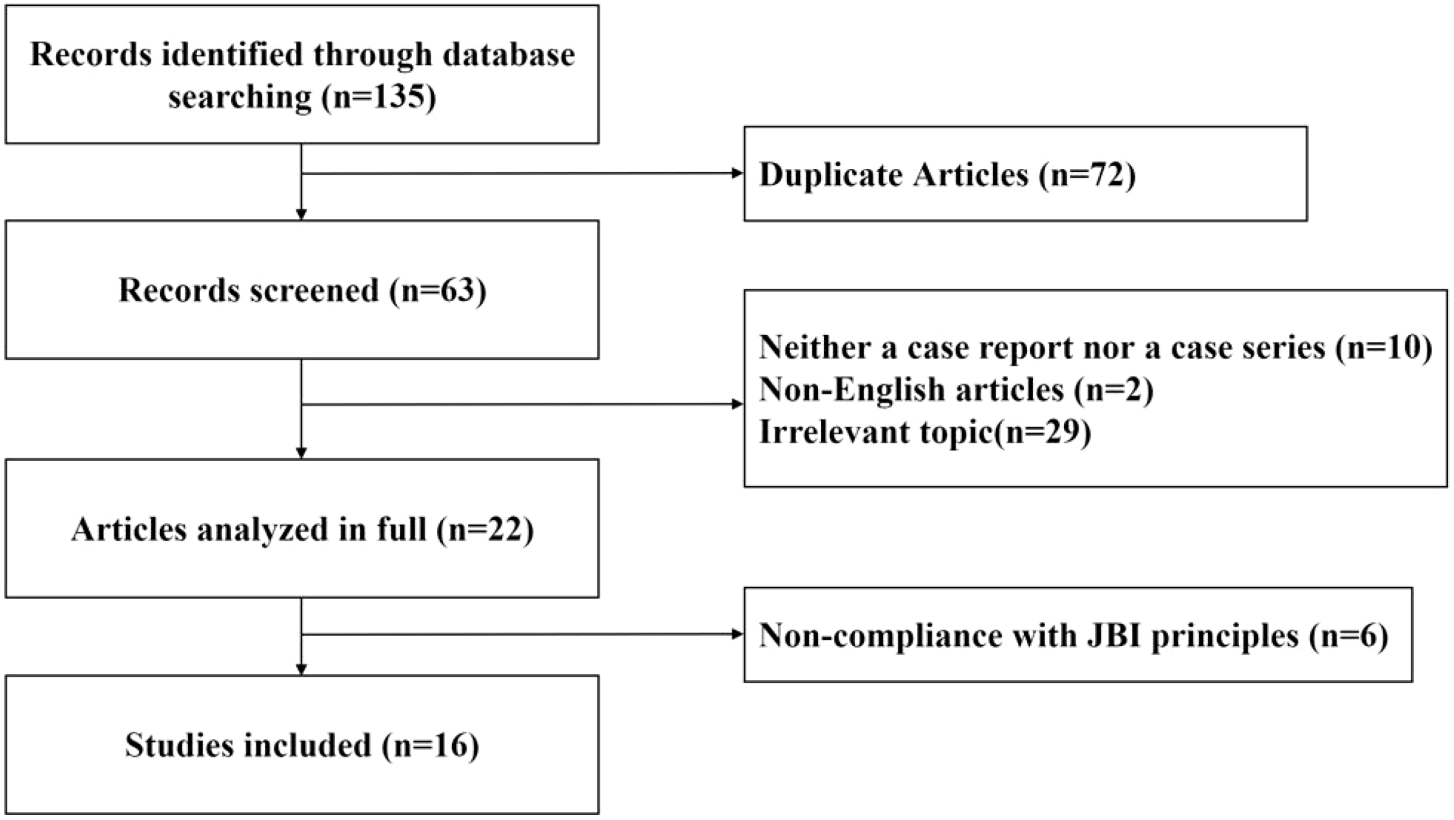
Study selection flow diagram.

A literature review was conducted to explore the effects of various auditory interventions on hearing outcomes in patients with CML experiencing hearing loss. Using the MeSH terms and keywords (“hearing loss” OR “sudden deafness”) AND (“CML” OR “chronic myeloid leukemia”), a comprehensive search was performed across PubMed, Web of Science, and Medline databases for case reports or case series published up to October 2024. The literature selection and evaluation were guided by the Joanna Briggs Institute (JBI) Critical Appraisal Checklist for case reports. Non-English articles, irrelevant topics, and studies that were neither case reports nor case series were excluded. Additionally, articles with less than 1 month of hearing follow-up showing no changes, as well as those not adhering to JBI principles, were excluded. A graphical depiction of the literature screening process is presented in **Figure 4**.

### Statistical analysis

The patient data included in the systematic review were analyzed using descriptive statistics.

## Results

Key data extracted from the papers selected for review on sudden hearing loss in CML cases are summarized in **Table 1**. In this review of case reports, the study cohort included 11 male and 5 female subjects, aged between 12 and 62 years. Among the patients, five presented with unilateral hearing loss (affecting the right ear in two cases and the left ear in three). Meanwhile, bilateral hearing loss was documented in 11 patients. 13 patients reported hearing loss as the initial symptom, whereas 3 patients experienced auditory decline following the onset of leukemia. Concurrent symptoms such as vertigo, tinnitus, and aural fullness were noted in 15 patients alongside hearing impairment.

**Table 1:** A summary of the case reports/series selected for systematic review.

Reference	Age	Sex	Side	Symptom Onset	Treatment	Cochlear Implant	Hearing Recovery	Pathogenesis	*WBC count (× 10^9^ cells/L)
Smith et al., 1991 (7)	62	M	B	Y	a	N	0	B	156
Hsu, Yao-Chung et al., 2004 (8)	35	M	L	Y	ac	N	0	A	151.2
Naithani, Rahul et al., 2005 (9)	12	F	B	Y	a	N	2	A	167
Cherchi, M et al., 2006 (10)	55	F	B	N	a	N	0	A	800
Acar, Gül Ozbilen et al.2007 (11)	50	M	R	Y	ac	N	0	A	567
Fatma Tulin Kayhan et al.2010 (12)	34	M	B	Y	ac	N	0	A	390.8
Gokce, Muge et al., 2010 (13)	15	F	R	Y	abc	N	2	A	455
Kapur, Sakshi et al., 2013 (14)	52	M	B	N	ad	Y	2	C	62.9
Diao, M et al.2014 (15)	31	F	L	Y	ac	N	0	A	264
Tang, Liyang et al., 2017 (16)	40	M	B	Y	bd	Y	2	C	697.1
Cass,Nathan D et al., 2018 (17)	49	M	B	Y	acd	N	1	A	555
Ren CHISHAKI et al., 2021 (18)	26	M	L	N	ac	N	0	A	627.3
Zeng, Xianhai et al., 2021 (19)	21	M	B	Y	ad	Y	2	A	626
Babakhanlou, Rodrick et al., 2023 (20)	23	M	B	Y	abc	N	1	B/C	81
Lahlou,G et al.2024 (21)	44	M	B	Y	abd	Y	2	A	795
Malhan,Hafiz et al., 2024 (22)	30	F	B	Y	ad	N	1	A	719.4

Age, the patient’s age at the onset of symptoms (one patient was described as a young person; however, their age was not specified); M, male; F, female; L, left-sided; R, right-sided; B, bilateral; Y, hearing loss as the initial symptom; N, hearing loss not as the initial symptom; A, hyperviscosity syndrome; B, leukemic infiltration of the temporal bone; C, associated with hemorrhagic lesions; a, leukoreduction therapy; b, Intrathecal chemotherapy; c, hormone therapy; d, Auditory assistance (include hearing aid and cochlear); Y, cochlear implant; N, without cochlear implantation; 0: No recovery described in the article. 1: Minor recovery described, or audiological tests suggest a recovery of 15–30 dB. 2: Significant recovery described, or audiological tests suggest a recovery of ≥30 dB; WBC, white blood cell. *Measured at the time of diagnosis, expressed as × 10^9^ cells/L.

The proposed mechanisms underlying hearing decline varied. Hyperviscosity syndrome was implicated in 12 cases (with 9 cases attributed to leukocytosis-induced labyrinthine artery infarction). In the remaining four patients, hearing loss was attributed to leukemic infiltration and hemorrhagic lesions.

Treatment regimens predominantly involved combination therapies, such as leukoreduction therapy (a), intrathecal chemotherapy (b), systemic or intratympanic glucocorticoid administration (c), and auditory assistance devices (d).In terms of auditory outcomes, seven patients showed no improvement, while nine demonstrated positive outcomes, categorized as mild (n = 3) or significant (n = 6) improvement. Among those with significant recovery, four had undergone cochlear implantation, one received intrathecal methotrexate, and one underwent leukoreduction therapy.

## Discussion

CML is a myeloproliferative disorder, which often arises when a hematopoietic stem cell acquires a translocation mutation between chromosomes 22 and 9, resulting in the fusion of the *BCR-ABL* oncogene. CML is characterized by the clonal proliferation of myeloid cells, leading to subsequent hyperleukocytosis, hyperviscosity syndrome, and leukocytosis (3). Hearing loss in patients with CML is very rare. To the best of our knowledge, only 16 cases of this phenomenon have been reported in the literature to date. The pathogenesis of CML-associated hearing loss is complex. Studies have revealed histopathologic alterations in the temporal bones, which can be divided into four primary categories: leukemic infiltration, leukostasis, hemorrhage of the cochlea or labyrinth, and infection (20). Recent reports favor leukostasis as the most likely etiology of acute otologic symptoms in patients with leukemia (9, 23). When leukocytosis occurs in CML, leukemic white blood cells are more likely to cause obstructive thrombi because they lose the plasticity required to traverse the microcirculation. Furthermore, because of their high oxygen demands, leukemic cells compete with normal white blood cells for this valuable resource in the microcirculation, which can lead to local tissue hypoxia and infarction (24, 25). The cochlea and vestibular apparatus receive their blood supply from the labyrinthine artery. Even a brief stasis can permanently damage the sensitive neuronal and vascular components of the inner ear (26). Given the extreme hyperleukocytosis (a WBC of 311 × 10^9^ cells/L) observed in our patient, a diagnosis of leukocytosis seems reasonable, and the damage caused to the cochlea, vascular or otherwise, appears permanent.

**Figure 5:**
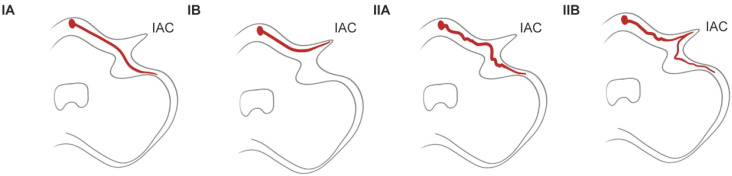
Anterior/posterior inferior cerebellar artery ranching patterns. IAC, internal auditory canal.

The anterior inferior cerebellar artery originates from the basilar artery and forms vascular collaterals close to the IAC opening. It then gives rise to the long, slender, tortuous internal auditory artery, which enters the IAC with the facial and auditory nerves to supply blood to the vestibule and cochlea (27). Many explanations have been proposed for the precise pathophysiology of sudden sensorineural hearing loss (SSHL) induced by vascular loops; these include direct nerve compression by the loop, nerve demyelination from contact with the artery, and nerve hypoxia arising from decreased perfusion (28, 29). In the present study, we used the Kazawa grading system (6), which is based on the 3D-FIESTA MRI findings, to describe the loop formation patterns of the AICA or posterior inferior cerebellar artery (PICA) branches and their extension into the IAC region. Type IA is characterized as non-looping AICA/PICA located in the CPA cistern, situated between the root entry zone (REZ) of the vestibular nerve and the orifice of the IAC. Type IB is identified as non-looping AICA/PICA that enters the IAC. Type IIA is classified as a loop-type AICA/PICA found in the CPA cistern, also positioned between the REZ of the vestibular nerve and the IAC orifice. Finally, Type IIB represents a loop-type AICA/PICA (**Table 2**, **Figure 5**). A previous study proposed that the loops of the type IIB branching pattern increase the cochlea’s susceptibility to ischemia (30). In our case, MRI showed that the left AICA assumed a type IIB branching pattern. The pathogenic mechanism of total deafness in the patient’s left ear was attributed to the marked increase in leukocyte numbers, which rendered the blood hyperviscous, leading to reduced microcirculatory blood flow, local tissue hypoxia, and infarction. The increased tortuosity of the vessels on the left side compared to the right reduces the blood supply from the labyrinthine artery to the left cochlea and vestibular organs. This reduced perfusion ultimately causes permanent damage to the sensitive neuronal and vascular components of the inner ear.

**Table 2:** MRI-based evaluation of AICA variations according to the Kazawa system.

Type	Branching Pattern
IA	Non-loop AICA/PICA in the cistern
IB	Non-loop AICA/PICA (distal AICA-internal auditory artery) entering the IAC
IIA	Loop type AICA/PICA in the CPA cistern
IIB	Loop type AICA/PICA entering the IAC

AICA, anterior inferior cerebellar artery; PICA, posterior inferior cerebellar artery; IAC, internal auditory canal; CPA, cerebellopontine angle.

The main treatment options for patients with CML-induced sudden deafness currently reported in the literature are the local and systemic administration of steroids, the used of nutritional neurological drugs, the intratympanic injection of methotrexate, leukapheresis, cochlear implantation, and hearing aids. These therapies are used in conjunction with aggressive treatment for CML. Although leukapheresis has a rapid cytoreductive effect, it is an intrusive technique, which does not address the underlying cause of CML. Thus, we do not routinely perform leukapheresis as a primary procedure in our hospital. Instead, our patient’s CML was targeted with imatinib mesylate, sodium bicarbonate, and allopurinol. After 22 days of treatment, the patient’s WBC count returned to normal (7.09 × 10^9^ cells/L).

Corticosteroids are a common treatment for SSNHL. Systemic steroids are typically administered for 5 days intravenously, while local steroid application is predominantly used as a salvage treatment. Post-aural steroid therapy is another widely used approach for localized drug delivery (31). Owing to the risk of secondary neurological damage associated with SSNHL pathogenesis, neurogenic drugs, such as mecobalamin, are also often considered. Most clinicians recognize ischemia of the cochlea, embolism, and vasospasm as key pathogenic mechanisms of SSNHL. Therefore, hemorheological forms of treatment, alongside fibrinogen reduction therapy with bacitracin, are commonly used to treat SSNHL in the clinic.

Cochlear implantation is a well-established method used to restore auditory function. According to the Clinical Practice Guideline: Sudden Hearing Loss (2019) (32), clinicians should conduct audiological assessments within 6 months following the initial diagnosis of SHL. In cases where auditory function fails to recover satisfactorily, cochlear implantation represents a potential therapeutic intervention. CML-associated cochlear ossification has rarely been reported. Labyrinthine artery occlusion and inner ear infection are recognized risk factors for cochlear ossification (33). Cochlear implantation is feasible in most patients with cochlear ossification (34); however, severe ossification of the cochlea renders cochlear implant surgery and cochleostomy more challenging (19).

Our patient received imatinib mesylate, sodium bicarbonate, and allopurinol for CML. Meanwhile, his hearing loss was targeted using systemic and local steroids(after excluding contraindication), hemoreological therapy, and nutritional neurological drugs. Unfortunately, although the patient’s CML was effectively controlled, his hearing could not be improved. We speculate, that, in this instance, hyperleukocytosis with leukocytosis and the obstruction of the labyrinthine and other minor arteries of the vertebrobasilar region most likely resulted in irreversible inner ear damage.

In conclusion, patients who experience sudden hearing loss as their first symptom should be thoroughly examined for fever, exhaustion, liver and spleen enlargement, and other symptoms. Blood testing should then be performed to rule out leukemia. Most cases of CML-associated hearing loss are thought to implicate hyperviscosity syndrome or labyrinthine artery occlusive infarction. If such a mechanism is suspected, the IAC can be visualized using MRI, and the patient can be trialed on thrombolytic drugs (batroxobin, etc.); however, this recommendation has not been validated. Future research needs to focus on improving the lives of individuals with leukemia-associated hearing loss, by placing more focus on the hearing outcome. Thus, we advocate for more aggressive cochlear implantation in patients who show no hearing recovery after 6 months of standard therapy, provided that the cochlea has not yet ossified and the leukemia is controlled sufficiently for surgical intervention.

## Data Availability

The original contributions presented in the study are included in the article/Supplementary Material. Further inquiries can be directed to the corresponding author/s.
